# 4-Hy­droxy-3-(3-meth­oxybenzoyl)-2-[(3-meth­oxybenzoyl)methyl]-2*H*-1,2-benzothia­zine 1,1-dioxide

**DOI:** 10.1107/S1600536810031673

**Published:** 2010-08-18

**Authors:** Salman Gul, Hamid Latif Siddiqui, Matloob Ahmad, Muhammad Nisar, Masood Parvez

**Affiliations:** a Institute of Chemistry, University of the Punjab, Lahore 54590, Pakistan; b Institute of Chemistry, University of the Punjab, Lahore-54590, Applied Chemistry Research Centre, PCSIR Laboratories Complex, Lahore 54600, Pakistan; c Institute of Chemical Sciences, University of Peshawar, Peshawar 25120, Pakistan; d Department of Chemistry, The University of Calgary, 2500 University Drive NW, Calgary, Alberta, Canada T2N 1N4

## Abstract

In the title compound, C_25_H_21_NO_7_S, the heterocyclic thia­zine ring adopts a half-chair conformation, with the S and N atoms displaced by −0.284 (3) and 0.411 (3) Å, respectively, from the plane formed by the remaining ring atoms; the puckering parameters are: *Q* = 0.4576 (13) Å, θ = 58.6 (2) and ϕ = 34.3 (3)°. The structure is devoid of any classical hydrogen bonds. However, intra­molecular C—H⋯N and O—H⋯O hydrogen bonds result in six-membered rings and inter­molecular C—H⋯O inter­actions stabilize the crystal structure.

## Related literature

For the biological applications of benzothia­zines, see: Lombardino *et al.* (1972 ▶); Zinnes *et al.* (1982 ▶); Zia-ur-Rehman *et al.* (2005 ▶); Turck *et al.* (1996 ▶); Ahmad *et al.* (2010 ▶). For related structures, see: Siddiqui *et al.* (2008 ▶). For puckering parameters, see: Cremer & Pople (1975 ▶).
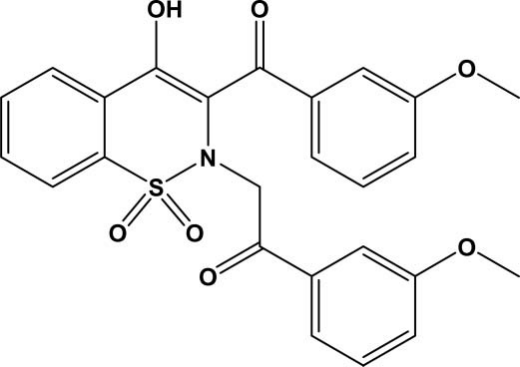


## Experimental

### 

#### Crystal data

C_25_H_21_NO_7_S
*M* *_r_* = 479.49
Triclinic, 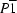

*a* = 10.3169 (2) Å
*b* = 10.6923 (3) Å
*c* = 11.6867 (3) Å
α = 115.5965 (11)°
β = 105.8041 (14)°
γ = 97.6128 (13)°
*V* = 1071.22 (5) Å^3^
*Z* = 2
Mo *K*α radiation
μ = 0.20 mm^−1^
*T* = 173 K
0.24 × 0.16 × 0.08 mm

#### Data collection

Nonius KappaCCD diffractometer
Absorption correction: multi-scan (*SORTAV*; Blessing, 1997 ▶) *T*_min_ = 0.953, *T*_max_ = 0.984
9164 measured reflections
4860 independent reflections
4419 reflections with (*I*) > 2.0 σ(*I*)
*R*_int_ = 0.021

#### Refinement

*R*[*F* ^2^ > 2σ(*F* ^2^)] = 0.038
*wR*(*F* ^2^) = 0.098
*S* = 1.07
4860 reflections
310 parameters
H-atom parameters constrained
Δρ_max_ = 0.37 e Å^−3^
Δρ_min_ = −0.42 e Å^−3^

### 

Data collection: *COLLECT* (Hooft, 1998 ▶); cell refinement: *HKL* *DENZO* (Otwinowski & Minor, 1997 ▶); data reduction: *SCALEPACK* (Otwinowski & Minor, 1997 ▶); program(s) used to solve structure: *SHELXS97* (Sheldrick, 2008 ▶); program(s) used to refine structure: *SHELXL97* (Sheldrick, 2008 ▶); molecular graphics: *ORTEP-3 for Windows* (Farrugia, 1997 ▶); software used to prepare material for publication: *SHELXL97*.

## Supplementary Material

Crystal structure: contains datablocks Global, I. DOI: 10.1107/S1600536810031673/jh2194sup1.cif

Structure factors: contains datablocks I. DOI: 10.1107/S1600536810031673/jh2194Isup2.hkl

Additional supplementary materials:  crystallographic information; 3D view; checkCIF report

## Figures and Tables

**Table 1:** Hydrogen-bond geometry (Å, °)

*D*—H⋯*A*	*D*—H	H⋯*A*	*D*⋯*A*	*D*—H⋯*A*
C25—H25*C*⋯O1^i^	0.98	2.57	3.438 (2)	147
C17—H17*B*⋯O2^i^	0.99	2.26	3.244 (2)	174
C15—H15⋯N1	0.95	2.41	2.986 (2)	119
O3—H3*O*⋯O4	0.84	1.67	2.428 (2)	149

Symmetry code: (i) 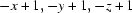.

**Fig. 1.**
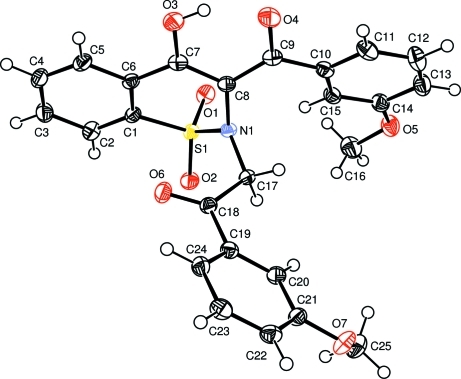
The title molecule plotted with the displacement ellipsoids at 50% probability level (Farrugia, 1997).

## supplementary crystallographic information

### Comment

Oxicams are non steroidal anti-inflammatory drugs (NSAID's) that posses benzothiazine nucleus (Lombardino *et al.*, 1972; Zinnes *et al.*, 1982). Versatile biological activities are associated with benzothiazine derivatives, *e.g.*, anti-microbial (Zia-ur-Rehman *et al.*, 2005), analgesic (Turck *et al.*, 1996), antioxidant (Ahmad *et al.*, 2010), *etc*. In this paper, we report the synthesis and crystal structure of the title compound.

The structure of the title compound contains independent molecules separated by normal van der Waals distances (Fig. 1). The heterocyclic thiazine ring adopts a half-chair conformation, with atoms S1 and N1 displaced by -0.284 (3) and 0.411 (3) Å, respectively, from the plane formed by atoms C1/C6/C7/C8; the puckering parameters (Cremer & Pople, 1975) are: *Q* = 0.4576 (13) Å, θ = 58.6 (2)° and φ = 34.3 (3)°. Similar conformations of the corresponding rings have been reported in some closely related compounds (Siddiqui *et al.*, 2008). The carbon fragments C1–C15 and C17–C24 are more or less planar individually and lie at an angle 77.17 (2)° with rest to each other.

The structure is devoid of any classical hydrogen bonds. However, intramolecular interactions C15—H15···N1 and O3—H3O···O4 resulting in six membered rings and intermolecular interactions of the type C—H···O are present (Tab. 1).

### Experimental

4-Hydroxy-1,1-dioxido-2*H*-1,2-benzothiazin-3-yl)(3-methoxyphenyl) methanone (2 g, 6.0 mmol), K_2_CO_3_ (1.24 g, 9.0 mmol), 3-methoxyphenacyl bromide (1.42 g, 6.2 mmol) and acetonitrile (25 ml) were refluxed for 6 h. The completion of reaction was monitored by TLC. After cooling to room temperature, the reaction mixture was poured into ice cold water. Yellow precipitates obtained were filtered, washed with cold water and dried. The crystals suitable for crystallographic study were grown from a solution of methanol and chloroform (1:1).

### Refinement

The H-atoms were located from difference Fourier maps and were included in the refinement at geometrically idealized positions in riding-model approximation with O—H = 0.84 Å and C—H = 0.95–0.99 Å; the *U*_iso_(H) were allowed at 1.2*U*_eq_(C) or 1.5*U*_eq_(O). The final difference map was essentially featurless.

### Crystal data

**Table:**

C_25_H_21_NO_7_S	*Z* = 2
*M**_r_* = 479.49	*F*(000) = 500
Triclinic, *P*1	*D*_x_ = 1.487 Mg m^−^^3^
Hall symbol: -P 1	Mo *K*α radiation, λ = 0.71073 Å
*a* = 10.3169 (2) Å	Cell parameters from 4699 reflections
*b* = 10.6923 (3) Å	θ = 1.0–27.5°
*c* = 11.6867 (3) Å	µ = 0.20 mm^−^^1^
α = 115.5965 (11)°	*T* = 173 K
β = 105.8041 (14)°	Prism, yellow
γ = 97.6128 (13)°	0.24 × 0.16 × 0.08 mm
*V* = 1071.22 (5) Å^3^	

### Data collection

**Table:**

Nonius KappaCCD diffractometer	4860 independent reflections
Radiation source: fine-focus sealed tube	4419 reflections with (*I*) > 2.0 σ(*I*)
graphite	*R*_int_ = 0.021
ω and φ scans	θ_max_ = 27.5°, θ_min_ = 2.1°
Absorption correction: multi-scan (*SORTAV*; Blessing, 1997)	*h* = −13→13
*T*_min_ = 0.953, *T*_max_ = 0.984	*k* = −13→13
9164 measured reflections	*l* = −15→15

### Refinement

**Table:**

Refinement on *F*^2^	Primary atom site location: structure-invariant direct methods
Least-squares matrix: full	Secondary atom site location: difference Fourier map
*R*[*F*^2^ > 2σ(*F*^2^)] = 0.038	Hydrogen site location: difference Fourier map
*wR*(*F*^2^) = 0.098	H-atom parameters constrained
*S* = 1.07	*w* = 1/[σ^2^(*F*_o_^2^) + (0.0352*P*)^2^ + 0.7607*P*] where *P* = (*F*_o_^2^ + 2*F*_c_^2^)/3
4860 reflections	(Δ/σ)_max_ < 0.001
310 parameters	Δρ_max_ = 0.37 e Å^−^^3^
0 restraints	Δρ_min_ = −0.42 e Å^−^^3^

### Special details

**Table:**

Experimental. Yield: 2.44 g, 85%, m.p. 434–435 K, IR (KBr, ν_max_): 2972, 1708, 1331, 1172 cm^-1^, EI—MS (*m/z*): 479.0
Geometry. All e.s.d.'s (except the e.s.d. in the dihedral angle between two l.s. planes) are estimated using the full covariance matrix. The cell e.s.d.'s are taken into account individually in the estimation of e.s.d.'s in distances, angles and torsion angles; correlations between e.s.d.'s in cell parameters are only used when they are defined by crystal symmetry. An approximate (isotropic) treatment of cell e.s.d.'s is used for estimating e.s.d.'s involving l.s. planes.
Refinement. Refinement of *F*^2^ against ALL reflections. The weighted *R*-factor *wR* and goodness of fit *S* are based on *F*^2^, conventional *R*-factors *R* are based on *F*, with *F* set to zero for negative *F*^2^. The threshold expression of *F*^2^ > σ(*F*^2^) is used only for calculating *R*-factors(gt) *etc*. and is not relevant to the choice of reflections for refinement. *R*-factors based on *F*^2^ are statistically about twice as large as those based on *F*, and *R*- factors based on ALL data will be even larger.

### Fractional atomic coordinates and isotropic or equivalent isotropic displacement parameters (Å^2^)

**Table:**

	*x*	*y*	*z*	*U*_iso_*/*U*_eq_	
S1	0.29020 (4)	0.27269 (4)	0.29750 (3)	0.01827 (10)	
O1	0.23063 (12)	0.17819 (13)	0.33899 (11)	0.0256 (2)	
O2	0.33929 (12)	0.42553 (12)	0.39028 (11)	0.0259 (2)	
O3	0.23349 (13)	−0.08669 (12)	−0.08736 (11)	0.0273 (3)	
H3O	0.2898	−0.1328	−0.0726	0.041*	
O4	0.42851 (13)	−0.14158 (13)	0.03455 (12)	0.0320 (3)	
O5	0.70078 (13)	0.17729 (14)	0.64535 (12)	0.0310 (3)	
O6	0.42789 (11)	0.38168 (13)	0.12184 (12)	0.0281 (3)	
O7	1.04913 (12)	0.66025 (14)	0.44194 (12)	0.0313 (3)	
N1	0.41973 (12)	0.22081 (13)	0.25537 (12)	0.0175 (2)	
C1	0.16599 (15)	0.23577 (16)	0.14140 (15)	0.0195 (3)	
C2	0.07019 (16)	0.31470 (18)	0.14134 (17)	0.0251 (3)	
H2	0.0773	0.3954	0.2236	0.030*	
C3	−0.03709 (17)	0.27353 (19)	0.01822 (18)	0.0277 (3)	
H3	−0.1043	0.3261	0.0163	0.033*	
C4	−0.04588 (16)	0.15639 (18)	−0.10117 (16)	0.0260 (3)	
H4	−0.1208	0.1274	−0.1842	0.031*	
C5	0.05357 (16)	0.08082 (17)	−0.10094 (15)	0.0231 (3)	
H5	0.0480	0.0024	−0.1840	0.028*	
C6	0.16194 (15)	0.11974 (16)	0.02119 (15)	0.0193 (3)	
C8	0.38133 (15)	0.07832 (15)	0.14093 (14)	0.0185 (3)	
C7	0.26316 (15)	0.03505 (16)	0.02289 (15)	0.0198 (3)	
C9	0.45739 (16)	−0.02239 (16)	0.14564 (15)	0.0219 (3)	
C10	0.56820 (16)	−0.00465 (16)	0.26834 (16)	0.0218 (3)	
C11	0.65958 (18)	−0.09010 (18)	0.24566 (18)	0.0278 (3)	
H11	0.6521	−0.1530	0.1552	0.033*	
C12	0.76153 (19)	−0.0826 (2)	0.35613 (19)	0.0337 (4)	
H12	0.8241	−0.1403	0.3408	0.040*	
C13	0.77297 (18)	0.0075 (2)	0.48786 (18)	0.0311 (4)	
H13	0.8431	0.0116	0.5626	0.037*	
C14	0.68140 (17)	0.09261 (17)	0.51133 (16)	0.0245 (3)	
C15	0.57875 (16)	0.08652 (16)	0.40222 (16)	0.0223 (3)	
H15	0.5159	0.1438	0.4180	0.027*	
C16	0.6187 (2)	0.2765 (2)	0.67580 (18)	0.0354 (4)	
H16A	0.6443	0.3321	0.7749	0.043*	
H16B	0.5186	0.2226	0.6324	0.043*	
H16C	0.6370	0.3429	0.6410	0.043*	
C17	0.54988 (15)	0.32707 (16)	0.29135 (15)	0.0194 (3)	
H17A	0.6254	0.2789	0.2869	0.023*	
H17B	0.5774	0.4045	0.3870	0.023*	
C18	0.54062 (15)	0.39652 (16)	0.20087 (15)	0.0194 (3)	
C19	0.67638 (15)	0.48054 (16)	0.21338 (15)	0.0199 (3)	
C20	0.79776 (16)	0.53606 (16)	0.33060 (15)	0.0216 (3)	
H20	0.7942	0.5258	0.4066	0.026*	
C21	0.92400 (15)	0.60661 (16)	0.33434 (16)	0.0222 (3)	
C22	0.92881 (17)	0.62253 (17)	0.22334 (17)	0.0250 (3)	
H22	1.0154	0.6691	0.2257	0.030*	
C23	0.80723 (17)	0.57047 (18)	0.10920 (17)	0.0263 (3)	
H23	0.8104	0.5839	0.0347	0.032*	
C24	0.68086 (16)	0.49892 (17)	0.10313 (16)	0.0233 (3)	
H24	0.5980	0.4627	0.0245	0.028*	
C25	1.04318 (18)	0.7108 (2)	0.57431 (17)	0.0323 (4)	
H25A	1.1368	0.7708	0.6436	0.039*	
H25B	1.0120	0.6279	0.5859	0.039*	
H25C	0.9767	0.7687	0.5845	0.039*	

### Atomic displacement parameters (Å^2^)

**Table:**

	*U*^11^	*U*^22^	*U*^33^	*U*^12^	*U*^13^	*U*^23^
S1	0.01845 (17)	0.01876 (18)	0.01474 (17)	0.00516 (13)	0.00559 (13)	0.00620 (14)
O1	0.0246 (5)	0.0319 (6)	0.0240 (6)	0.0064 (5)	0.0112 (4)	0.0160 (5)
O2	0.0275 (6)	0.0205 (6)	0.0193 (5)	0.0081 (4)	0.0047 (4)	0.0030 (4)
O3	0.0335 (6)	0.0221 (6)	0.0177 (5)	0.0075 (5)	0.0065 (5)	0.0045 (5)
O4	0.0389 (7)	0.0217 (6)	0.0249 (6)	0.0131 (5)	0.0092 (5)	0.0028 (5)
O5	0.0354 (6)	0.0343 (7)	0.0230 (6)	0.0139 (5)	0.0076 (5)	0.0147 (5)
O6	0.0193 (5)	0.0358 (7)	0.0315 (6)	0.0052 (5)	0.0049 (5)	0.0220 (5)
O7	0.0183 (5)	0.0408 (7)	0.0251 (6)	0.0006 (5)	0.0046 (5)	0.0123 (5)
N1	0.0169 (6)	0.0149 (6)	0.0167 (6)	0.0026 (4)	0.0053 (5)	0.0056 (5)
C1	0.0163 (6)	0.0218 (7)	0.0184 (7)	0.0026 (5)	0.0047 (5)	0.0101 (6)
C2	0.0231 (7)	0.0268 (8)	0.0246 (8)	0.0079 (6)	0.0087 (6)	0.0117 (7)
C3	0.0218 (7)	0.0316 (9)	0.0335 (9)	0.0092 (6)	0.0082 (7)	0.0200 (7)
C4	0.0201 (7)	0.0302 (8)	0.0246 (8)	0.0003 (6)	0.0017 (6)	0.0166 (7)
C5	0.0225 (7)	0.0236 (8)	0.0176 (7)	−0.0006 (6)	0.0041 (6)	0.0093 (6)
C6	0.0187 (7)	0.0188 (7)	0.0179 (7)	0.0007 (5)	0.0052 (5)	0.0091 (6)
C8	0.0202 (7)	0.0164 (7)	0.0174 (7)	0.0035 (5)	0.0080 (5)	0.0068 (6)
C7	0.0231 (7)	0.0180 (7)	0.0169 (7)	0.0028 (6)	0.0085 (6)	0.0077 (6)
C9	0.0241 (7)	0.0189 (7)	0.0212 (7)	0.0043 (6)	0.0093 (6)	0.0084 (6)
C10	0.0236 (7)	0.0190 (7)	0.0257 (8)	0.0066 (6)	0.0101 (6)	0.0126 (6)
C11	0.0347 (9)	0.0244 (8)	0.0300 (8)	0.0136 (7)	0.0163 (7)	0.0141 (7)
C12	0.0360 (9)	0.0369 (10)	0.0411 (10)	0.0229 (8)	0.0188 (8)	0.0237 (8)
C13	0.0301 (9)	0.0352 (9)	0.0335 (9)	0.0148 (7)	0.0087 (7)	0.0218 (8)
C14	0.0261 (8)	0.0243 (8)	0.0262 (8)	0.0070 (6)	0.0095 (6)	0.0151 (7)
C15	0.0236 (7)	0.0208 (7)	0.0260 (8)	0.0081 (6)	0.0095 (6)	0.0137 (6)
C16	0.0469 (11)	0.0305 (9)	0.0256 (8)	0.0165 (8)	0.0121 (8)	0.0103 (7)
C17	0.0167 (6)	0.0182 (7)	0.0192 (7)	0.0012 (5)	0.0037 (5)	0.0085 (6)
C18	0.0193 (7)	0.0175 (7)	0.0190 (7)	0.0049 (5)	0.0060 (5)	0.0076 (6)
C19	0.0197 (7)	0.0177 (7)	0.0227 (7)	0.0056 (5)	0.0085 (6)	0.0099 (6)
C20	0.0217 (7)	0.0208 (7)	0.0210 (7)	0.0048 (6)	0.0074 (6)	0.0099 (6)
C21	0.0191 (7)	0.0197 (7)	0.0238 (7)	0.0046 (6)	0.0065 (6)	0.0086 (6)
C22	0.0244 (8)	0.0224 (7)	0.0302 (8)	0.0053 (6)	0.0129 (6)	0.0134 (7)
C23	0.0306 (8)	0.0274 (8)	0.0276 (8)	0.0084 (7)	0.0144 (7)	0.0169 (7)
C24	0.0236 (7)	0.0245 (8)	0.0230 (7)	0.0071 (6)	0.0074 (6)	0.0133 (6)
C25	0.0263 (8)	0.0363 (9)	0.0233 (8)	0.0068 (7)	0.0041 (6)	0.0090 (7)

### Geometric parameters (Å, °)

**Table:**

S1—O2	1.4329 (11)	C10—C15	1.404 (2)
S1—O1	1.4334 (11)	C11—C12	1.389 (2)
S1—N1	1.6294 (12)	C11—H11	0.9500
S1—C1	1.7597 (15)	C12—C13	1.379 (3)
O3—C7	1.2956 (18)	C12—H12	0.9500
O3—H3O	0.8400	C13—C14	1.396 (2)
O4—C9	1.2875 (19)	C13—H13	0.9500
O5—C14	1.367 (2)	C14—C15	1.388 (2)
O5—C16	1.430 (2)	C15—H15	0.9500
O6—C18	1.2126 (18)	C16—H16A	0.9800
O7—C21	1.3686 (18)	C16—H16B	0.9800
O7—C25	1.424 (2)	C16—H16C	0.9800
N1—C8	1.4344 (18)	C17—C18	1.523 (2)
N1—C17	1.4651 (17)	C17—H17A	0.9900
C1—C2	1.382 (2)	C17—H17B	0.9900
C1—C6	1.401 (2)	C18—C19	1.494 (2)
C2—C3	1.395 (2)	C19—C24	1.396 (2)
C2—H2	0.9500	C19—C20	1.401 (2)
C3—C4	1.384 (2)	C20—C21	1.396 (2)
C3—H3	0.9500	C20—H20	0.9500
C4—C5	1.388 (2)	C21—C22	1.392 (2)
C4—H4	0.9500	C22—C23	1.387 (2)
C5—C6	1.398 (2)	C22—H22	0.9500
C5—H5	0.9500	C23—C24	1.389 (2)
C6—C7	1.472 (2)	C23—H23	0.9500
C8—C7	1.409 (2)	C24—H24	0.9500
C8—C9	1.425 (2)	C25—H25A	0.9800
C9—C10	1.488 (2)	C25—H25B	0.9800
C10—C11	1.396 (2)	C25—H25C	0.9800
			
O2—S1—O1	118.83 (7)	C12—C13—C14	119.97 (16)
O2—S1—N1	108.34 (7)	C12—C13—H13	120.0
O1—S1—N1	108.04 (7)	C14—C13—H13	120.0
O2—S1—C1	110.07 (7)	O5—C14—C15	124.51 (15)
O1—S1—C1	107.34 (7)	O5—C14—C13	115.40 (14)
N1—S1—C1	103.07 (7)	C15—C14—C13	120.08 (15)
C7—O3—H3O	109.5	C14—C15—C10	119.71 (14)
C14—O5—C16	117.64 (13)	C14—C15—H15	120.1
C21—O7—C25	117.12 (13)	C10—C15—H15	120.1
C8—N1—C17	119.25 (11)	O5—C16—H16A	109.5
C8—N1—S1	115.44 (9)	O5—C16—H16B	109.5
C17—N1—S1	120.85 (10)	H16A—C16—H16B	109.5
C2—C1—C6	122.10 (14)	O5—C16—H16C	109.5
C2—C1—S1	119.57 (12)	H16A—C16—H16C	109.5
C6—C1—S1	118.19 (11)	H16B—C16—H16C	109.5
C1—C2—C3	118.73 (15)	N1—C17—C18	114.76 (12)
C1—C2—H2	120.6	N1—C17—H17A	108.6
C3—C2—H2	120.6	C18—C17—H17A	108.6
C4—C3—C2	120.18 (15)	N1—C17—H17B	108.6
C4—C3—H3	119.9	C18—C17—H17B	108.6
C2—C3—H3	119.9	H17A—C17—H17B	107.6
C3—C4—C5	120.69 (14)	O6—C18—C19	122.43 (14)
C3—C4—H4	119.7	O6—C18—C17	120.85 (13)
C5—C4—H4	119.7	C19—C18—C17	116.71 (12)
C4—C5—C6	120.20 (15)	C24—C19—C20	120.41 (14)
C4—C5—H5	119.9	C24—C19—C18	118.28 (13)
C6—C5—H5	119.9	C20—C19—C18	121.27 (13)
C5—C6—C1	118.03 (14)	C21—C20—C19	119.16 (14)
C5—C6—C7	120.31 (14)	C21—C20—H20	120.4
C1—C6—C7	121.57 (13)	C19—C20—H20	120.4
C7—C8—C9	119.14 (13)	O7—C21—C22	115.97 (14)
C7—C8—N1	119.46 (13)	O7—C21—C20	123.67 (14)
C9—C8—N1	121.37 (13)	C22—C21—C20	120.34 (14)
O3—C7—C8	121.27 (14)	C23—C22—C21	120.02 (14)
O3—C7—C6	116.54 (13)	C23—C22—H22	120.0
C8—C7—C6	122.05 (13)	C21—C22—H22	120.0
O4—C9—C8	118.14 (14)	C22—C23—C24	120.47 (15)
O4—C9—C10	115.58 (14)	C22—C23—H23	119.8
C8—C9—C10	126.27 (14)	C24—C23—H23	119.8
C11—C10—C15	119.87 (14)	C23—C24—C19	119.57 (14)
C11—C10—C9	116.94 (14)	C23—C24—H24	120.2
C15—C10—C9	123.09 (14)	C19—C24—H24	120.2
C12—C11—C10	119.58 (16)	O7—C25—H25A	109.5
C12—C11—H11	120.2	O7—C25—H25B	109.5
C10—C11—H11	120.2	H25A—C25—H25B	109.5
C13—C12—C11	120.78 (16)	O7—C25—H25C	109.5
C13—C12—H12	119.6	H25A—C25—H25C	109.5
C11—C12—H12	119.6	H25B—C25—H25C	109.5
			
O2—S1—N1—C8	−166.79 (10)	N1—C8—C9—C10	8.9 (2)
O1—S1—N1—C8	63.25 (12)	O4—C9—C10—C11	20.3 (2)
C1—S1—N1—C8	−50.17 (12)	C8—C9—C10—C11	−160.34 (15)
O2—S1—N1—C17	−10.66 (13)	O4—C9—C10—C15	−156.17 (15)
O1—S1—N1—C17	−140.62 (11)	C8—C9—C10—C15	23.2 (2)
C1—S1—N1—C17	105.97 (11)	C15—C10—C11—C12	−0.9 (2)
O2—S1—C1—C2	−38.43 (14)	C9—C10—C11—C12	−177.40 (15)
O1—S1—C1—C2	92.25 (13)	C10—C11—C12—C13	0.4 (3)
N1—S1—C1—C2	−153.82 (12)	C11—C12—C13—C14	0.0 (3)
O2—S1—C1—C6	145.82 (11)	C16—O5—C14—C15	−6.3 (2)
O1—S1—C1—C6	−83.50 (12)	C16—O5—C14—C13	174.28 (15)
N1—S1—C1—C6	30.43 (13)	C12—C13—C14—O5	179.53 (16)
C6—C1—C2—C3	2.6 (2)	C12—C13—C14—C15	0.1 (3)
S1—C1—C2—C3	−172.99 (12)	O5—C14—C15—C10	−179.92 (14)
C1—C2—C3—C4	−0.5 (2)	C13—C14—C15—C10	−0.5 (2)
C2—C3—C4—C5	−1.7 (2)	C11—C10—C15—C14	0.9 (2)
C3—C4—C5—C6	1.9 (2)	C9—C10—C15—C14	177.24 (14)
C4—C5—C6—C1	0.2 (2)	C8—N1—C17—C18	77.60 (17)
C4—C5—C6—C7	176.77 (13)	S1—N1—C17—C18	−77.64 (15)
C2—C1—C6—C5	−2.4 (2)	N1—C17—C18—O6	11.8 (2)
S1—C1—C6—C5	173.19 (11)	N1—C17—C18—C19	−167.24 (12)
C2—C1—C6—C7	−178.99 (14)	O6—C18—C19—C24	−22.8 (2)
S1—C1—C6—C7	−3.36 (19)	C17—C18—C19—C24	156.26 (14)
C17—N1—C8—C7	−111.52 (15)	O6—C18—C19—C20	159.16 (15)
S1—N1—C8—C7	45.01 (16)	C17—C18—C19—C20	−21.8 (2)
C17—N1—C8—C9	70.61 (18)	C24—C19—C20—C21	−1.7 (2)
S1—N1—C8—C9	−132.85 (12)	C18—C19—C20—C21	176.33 (14)
C9—C8—C7—O3	−9.2 (2)	C25—O7—C21—C22	150.29 (15)
N1—C8—C7—O3	172.85 (13)	C25—O7—C21—C20	−31.4 (2)
C9—C8—C7—C6	166.26 (13)	C19—C20—C21—O7	−177.72 (14)
N1—C8—C7—C6	−11.6 (2)	C19—C20—C21—C22	0.5 (2)
C5—C6—C7—O3	−10.3 (2)	O7—C21—C22—C23	179.53 (15)
C1—C6—C7—O3	166.13 (13)	C20—C21—C22—C23	1.2 (2)
C5—C6—C7—C8	173.95 (13)	C21—C22—C23—C24	−1.7 (2)
C1—C6—C7—C8	−9.6 (2)	C22—C23—C24—C19	0.5 (2)
C7—C8—C9—O4	10.4 (2)	C20—C19—C24—C23	1.2 (2)
N1—C8—C9—O4	−171.74 (13)	C18—C19—C24—C23	−176.90 (14)
C7—C8—C9—C10	−169.00 (14)		

### Hydrogen-bond geometry (Å, °)

**Table:**

*D*—H···*A*	*D*—H	H···*A*	*D*···*A*	*D*—H···*A*
C25—H25C···O1^i^	0.98	2.57	3.438 (2)	147
C17—H17B···O2^i^	0.99	2.26	3.244 (2)	174
C17—H17B···O2	0.99	2.51	2.844 (2)	100
C15—H15···N1	0.95	2.41	2.986 (2)	119
O3—H3O···O4	0.84	1.67	2.428 (2)	149

Symmetry codes: (i) −*x*+1, −*y*+1, −*z*+1.
